# Altered milk tryptophan and tryptophan metabolites and health of children born to women with HIV

**DOI:** 10.21203/rs.3.rs-6229815/v1

**Published:** 2025-03-21

**Authors:** Nicole H. Tobin, Fan Li, Wentao Zhu, Kathie G. Ferbas, John W. Sleasman, Daniel Raftery, Louise Kuhn, Grace M. Aldrovandi

**Affiliations:** 1 Division of InfecGous Diseases, Department of Pediatrics, David Geffen School of Medicine at the University of California, Los Angeles; 2 UW Northwest Metabolomics Center; 3 Division of Pediatric Allergy and Immunology, Department of Pediatrics, Duke University School of Medicine, Durham, NC.; 4 Gertrude H. Sergievsky Center, Vagelos College of Physicians and Surgeons; and Department of Epidemiology, Mailman School of Public Health, Columbia University Irving Medical Center, New York, NY

**Keywords:** CHEU, HEU, milk, human milk, breastmilk, HIV, WWH, Tryptophan, Kynurenine, Viperin, ddhC, ddhCTP, dimethylarginine, cytosine, interferon-inducible cytokines, type I interferons

## Abstract

Children born to women with HIV (WWH) suffer increased morbidity and, in low-income settings, have two to three times the mortality of infants born to women without HIV. The basis for this increase remains elusive. In low-income settings, breastfeeding is recommended because health benefits outweigh the risk of transmission, especially when maternal antiretroviral therapy is provided. We profiled the milk metabolome of 326 women sampled longitudinally for 18 months postpartum using global metabolomics. We identify perturbations in several metabolites, including tryptophan, dimethylarginine, and a recently discovered antiviral ribonucleotide, that are robustly associated with maternal HIV infection. Quantitative tryptophan and kynurenine levels in both milk and plasma reveal that these perturbations reflect systemic depletion of tryptophan and alterations in tryptophan catabolism in WWH. Our findings provide intriguing evidence that decreases in tryptophan availability and perturbations in tryptophan catabolism in children born to WWH may contribute to their increased morbidity and mortality.

## Introduction

Children born to women with HIV (WWH) experience significantly increased morbidity and mortality, even when they themselves are not infected (HIV-exposed, or CHEU). While CHEU in high income countries are more likely to be hospitalized^1–3^, those in low-income countries have mortality rates two to three times higher than infants born to women without HIV (WWoH)^4–7^. Most of this morbidity and mortality is due to infections, particularly pneumonia, diarrhea, and meningitis^7,8^. Additionally, CHEU face challenges related to growth and cognitive development^7^. Although maternal antiretroviral therapy has improved CHEU health outcomes, they still experience significant risks, the basis of which remains unclear.

HIV infection, even with optimal viral suppression by antiretroviral therapy, is associated with chronic inflammation characterized by persistent macrophage activation, elevated levels of interferon inducible cytokines, and intestinal barrier dysfunction^9^. This systemic inflammation also affects pregnant WWH and is reflected in their CHEU infants^10–14^. Similar to their mothers, CHEU infants display perturbed biomarkers of immune monocyte activation, including elevated interferon-inducible cytokines, which impair immune function and germinal center development throughout infancy^15–18^. Such changes are not unique to HIV as maternal immune activation due to maternal influenza and other infections is associated with the development of neurodevelopmental disorders^19–24^ as well as the development of tissue specific immunity and inflammation^25–27^ in their offspring.

HIV-associated immune dysregulation is largely driven by metabolic alterations, particularly in the indoleamine 2,3-dioxygenase (IDO) pathway and accelerated tryptophan catabolism, both of which impair immune function.^28,29^ Tryptophan, an essential amino acid obtained solely from the diet, is primarily catabolized via the kynurenine pathway, with its metabolites playing key roles in immune regulation, neuronal function, and intestinal homeostasis^30–32^. A key marker of IDO activation is the kynurenine-to-tryptophan (KT) ratio^33^, which is elevated in HIV infection and associated with disease progression, dementia, and mortality^34^. Although the KT ratio decreases with viral suppression, it does not normalize completely^35,36^. High KT ratios are linked to microbial dysbiosis, TH17 cell loss, and an imbalance between TH17 and Treg cells in the gastrointestinal mucosa^29,37^. Additionally, elevated KT ratios have been associated with stunting in children^38,39^, suggesting that disrupted tryptophan metabolism may have broader health impacts.

**Figure 1:**
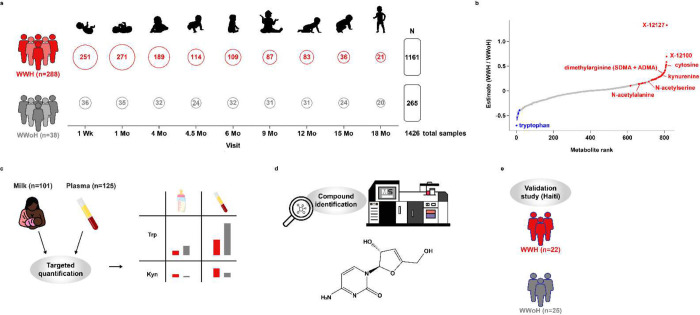
Study Design. a) Large, global metabolomic study of milk of WWH versus WWoH. Open circles denote number of samples at each study visit included in the final analysis. The number of participants in each group is indicated in parentheses on the left, and the number of samples is indicated along the right. b) Differentially abundant metabolites in the milk WWH vs WWoH, shown in rank order. c) Targeted quantification of tryptophan and kynurenine levels in maternal milk and plasma. d) Identification of the unknown compound X-12127 as 3’-deoxy-3’,4’-didehydro-cytidine (ddhC). e) Validation of milk metabolomic findings in an independent cohort of WWH from the Haiti study.

Breast milk is essential for optimal growth and development, particularly for infants born to WWH in low- and middle-income countries. The dietary source of tryptophan for nursing infants is human milk. Utilizing longitudinal samples from a cohort of WWH and WWoH from the pre-antiretroviral therapy era, we characterize the metabolomic profile of breast milk over the first 18 months postpartum. We identify a metabolomic signature of milk in WWH that demonstrates decreased levels of tryptophan throughout lactation. This depletion of tryptophan is associated with elevations of the type I interferon response^28^. Using targeted assays for tryptophan and kynurenine in both milk and plasma, we demonstrate that quantitative levels of tryptophan and kynurenine are systemically altered in WWH. Furthermore, in later experiments we identify an initially unknown compound, which is highly elevated in the milk of WWH, as 3’-deoxy-3’4’-didehydro-cytidine (ddhC), the free base of an interferon-inducible innate antiviral ribonucleotide^40^. Lastly, we confirm this milk metabolomic signature of HIV in a second, healthier cohort of WWH on antiretroviral therapy (see study overview, Figure 1).

## Results

### More than eight-hundred metabolites characterized over 18 months of lactation in 326 women

To investigate the milk metabolome of WWH, global metabolomics was performed on 1599 whole human milk samples collected as part of a randomized clinical trial^41^. The Zambia Exclusive Breastfeeding Study (ZEBS) was conducted in Lusaka, Zambia between 2001 and 2008 and investigated the feeding strategy for WWH recommended by the World Health Organization at that time. This trial was conducted prior to the routine use of antiretroviral therapy with most women receiving single dose nevirapine for prevention of vertical transmission per standard of care. Women/infant pairs were followed for 24 months postpartum. Longitudinal breast milk samples spanning the first 24 months of lactation were selected from 38 WWoH and a stratified sample of 288 WWH enrolled in the trial. The clinical characteristics of the study participants are shown in Table 1. Thirty-six (12.5%) of WWH died during the course of the study compared to no WWoH (p<0.05). WWH had lower CD4 T cell counts (315 vs. 840 cells/mm^3^) and higher CD8 T cell counts (759 vs 563 cells/mm^3^) than WWoH (p<0.001 and p=0.001, respectively). Children of WWoH weighed more at birth (3.3 vs 2.9 kg; p<0.01) and 3 of these children died during the study.

All but 2 of the 1599 whole breast milk samples were successfully characterized and included 765 named and 74 unnamed metabolites for a total of 839 metabolites. Of the 765 named metabolites, 20 metabolites classified as “drug-antibiotic” or “drug-antiviral” were removed from further analyses, leaving a total of 819 metabolites included in the analyses. Samples were excluded from this analysis if there were insufficient samples at that timepoint for meaningful interpretation (n=72) or if the milk was collected post-weaning (n=99), leaving 1,426 samples spanning 18 months of lactation in the final analysis (Figure 1A).

### Metabolomic signature of maternal HIV infection in milk

**Figure 2:**
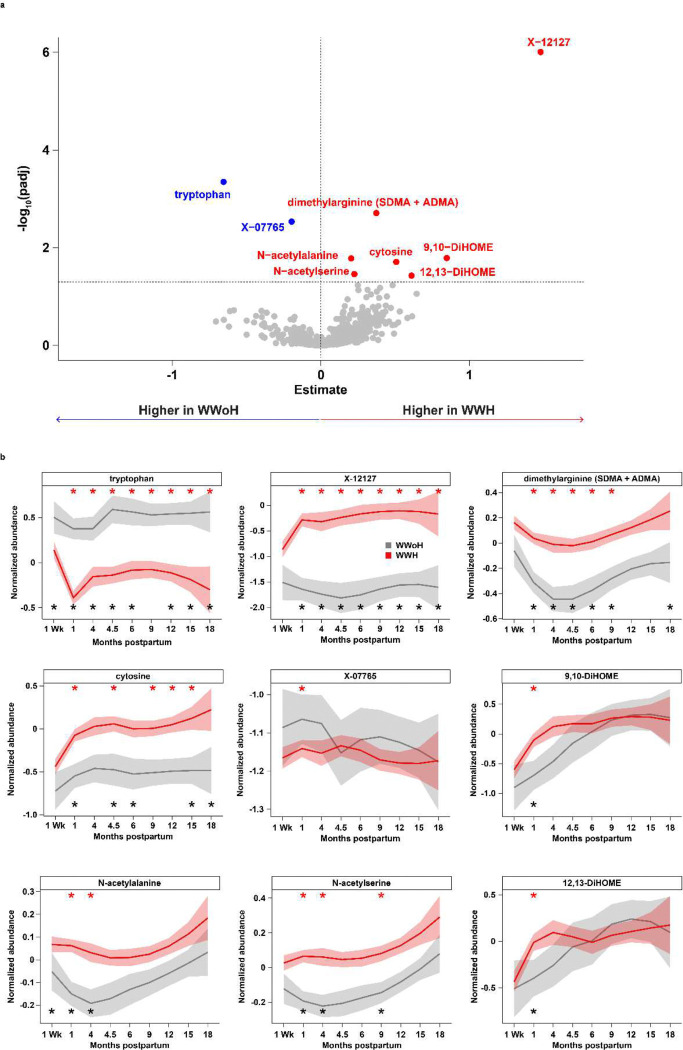
Metabolomic signature of HIV infection in milk. a) Volcano plot showing differences in metabolite abundances at the 1-month timepoint. Blue and red points metabolites are significantly decreased and increased in WWH versus WWoH, respectively. b) Normalized abundances of selected metabolites across the study course. Solid lines indicate mean abundances, and shaded areas denote 95% confidence intervals. Red asterisks along the top denote study visits at which the selected compound was differentially abundant in WWH versus WWoH. Black asterisks along the bottom denote study visits at which the compound was selected as a predictive feature in the random forests modeling. All metabolites that were significantly differentially abundant at the 1-month timepoint are shown.

Overall milk metabolomic profiles were strongly influenced by study visit (4.0% variance, p<0.001), and by HIV infection (0.2% variance, p<0.001), maternal CD4 count (0.15% variance, p<0.001), and infant sex (0.07% variance, p=0.009) (**Extended Data Fig. 1**). Linear mixed effects regression identified 173 metabolites with significantly altered levels in WWH versus WWoH when all study visits were considered in aggregate (**Extended Data Table 1**). Post hoc comparisons stratified by study visit identified a core set of altered metabolites (Figures 2A and **Extended Data Fig. 2; Extended Data Table 2**), including tryptophan, dimethylarginine, cytosine, and an uncharacterized compound, X-12127. Levels of tryptophan, dimethylarginine, cytosine, and X-12127 were significantly and consistently altered in the milk of WWH across the entire study time course (p <0.001; Figure 2B, red stars).

As expected, tryptophan, cytosine and X-12127 were strongly correlated with baseline maternal CD4 count, baseline maternal plasma viral load, and contemporaneous breast milk viral load (**Extended Data Fig. 3 and Extended Data Table 3**). N-acetylalanine (p = 0.006) and N-acetylserine (p = 0.007) were elevated in WWH with greater separation at the earlier timepoints, 1 and 4 months. In contrast, the linoleic acid metabolites 9,10-DiHOME and 12,13-DiHOME were significantly elevated in WWH at 1 month (p < 0.05), but not over the entire study time course. X-07765, an unknown metabolite significantly elevated at the one-month timepoint in the linear regression model (p = 0.003), did not appear to be as robust a marker as the other metabolites and was not significant over the entire time course (p = NS; Figure 2B). In an orthogonal analysis, random forests models for maternal HIV status also identified many of these same features as significant predictors of maternal HIV (Figure 2B black stars, **Extended Data Fig. 4, Extended Data Table 4**). Finally, a sub-analysis of WWH whose children remained uninfected during the course of the study (38 WWoH versus 154 WWH with CHEU) confirmed the metabolic signature of maternal HIV infection to be independent of child outcome (**Extended Data Fig. 5 and Extended Data Tables 5 and 6**). Taken together, these results point to a robust and sustained effect of HIV infection on the composition of metabolically relevant compounds in human milk.

### Alteration in tryptophan and its metabolites in the milk of WWH

The striking separation in normalized abundances of tryptophan and X-12127 across the study course (Figure 2B) led to further investigations of these two metabolites. Of note, although tryptophan abundance declined slightly between the 1 week and 1 month milk samples in WWoH, there was a notable dip in tryptophan abundance in the WWH followed by some recovery and continued significant separation of tryptophan throughout the study course. The normalized abundance of X-12127 rose sharply between 1 week and 1 month and remained elevated, a pattern that was mirrored by cytosine. It was also noted that dimethylarginine, N-acetylalanine and N-acetylserine all had similarly shaped normalized abundance curves.

**Figure 3:**
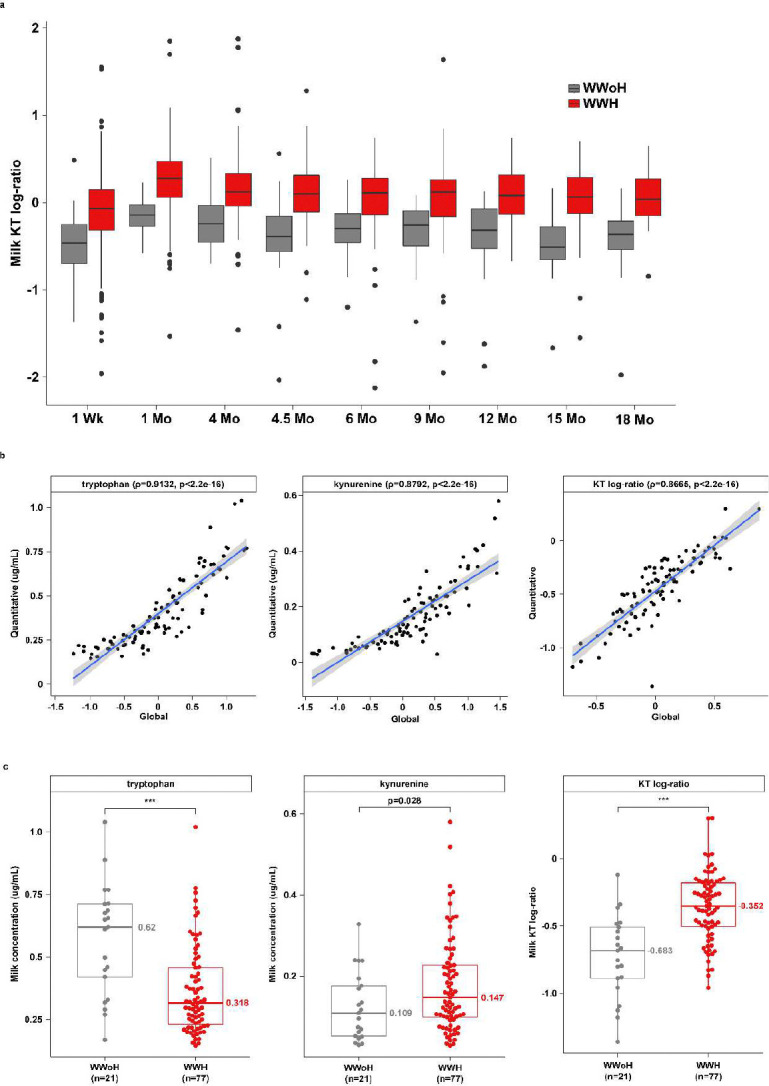
Altered tryptophan and kynurenine levels in the milk of WWH. a) Boxplot of kynurenine/tryptophan log-ratio in WWH versus WWoH stratified by study visit, as calculated from the global metabolomics panel (n=1426; p < 0.001 all timepoints). b) Scatterplots of global versus quantitative tryptophan and kynurenine abundances and KT log-ratio at the 4-month timepoint (n=98). Solid blue line indicates linear regression line and shaded gray area represents 95% confidence intervals. Spearman correlation coefficients and p-values are shown in parentheses in each subtitle. c) Tryptophan, kynurenine, and KT log-ratio values in WWH versus WWoH at the 4-month study visit, as calculated from the quantitative panel. * p < 0.05, ** p < 0.01, *** p < 0.001. WWH: Women with HIV; WWoH: Women without HIV.

The significant depletion of tryptophan across the study duration was mirrored by an increase in levels of X-12100 (tentatively identified as a hydroxylated tryptophan compound) as well as a consistent, albeit smaller, increase in levels of kynurenine (**Extended Data Fig. 6 and Extended Data Tables 1**-2). Although none of the comparisons in the analysis stratified by visit demonstrated significant differences after adjustment for multiple comparisons (**Extended Data Table 2**), kynurenine was increased in the mean effects model (p=0.007, **Extended Data Table 1**). Moreover, the kynurenine to tryptophan (KT) ratio, a marker of IDO activity, was significantly elevated at all study visits (Figure 3A and **Extended Data Table 7**; p < 0.001).

To confirm the findings of the elevated KT ratio from the global metabolomics panel, a quantitative KT panel was performed on 98 milk samples from 21 WWoH and 77 WWH at the 4-month timepoint. The 4-month timepoint was selected because paired milk and plasma samples were available from almost all participants at this timepoint due to the trial design. Quantitative and global KT values were highly correlated (Figure 3B, rho=0.9132 and 0.8792 for tryptophan and kynurenine, respectively; both p<2.2e-16), indicating that the global panel could be used for accurate estimation of the quantitative KT-ratio. Milk tryptophan levels were significantly lower, 0.318 ug/mL versus 0.62 ug/mL (p<0.001), and kynurenine levels were significantly higher, 0.147 ug/mL versus 0.109 ug/mL (p=0.028) in WWH versus WWoH (Figure 3C and **Extended Data Table 8**). Correspondingly, the KT log-ratio was significantly higher in WWH as well (p<0.001, Figure 3C and **Extended Data Table 8**).

### Lower milk tryptophan levels mirror lower plasma tryptophan levels

The lower levels of tryptophan in milk of WWH could reflect lower plasma levels of tryptophan in WWH, decreased transfer of tryptophan to the milk of WWH or altered tryptophan metabolism in the milk of WWH. Given that tryptophan transport across the gut can be perturbed by infection^42^, we sought to determine whether transport across the breast epithelium was similarly affected. To distinguish between these possibilities, quantitative plasma and milk tryptophan and kynurenine levels were compared from 118 women (31 WWoH and 87 WWH). As expected, milk and plasma levels were highly correlated (rho=0.50, p<0.001 for kynurenine; rho=0.66, p<0.001 for tryptophan). Plasma tryptophan levels were significantly lower, 5.11 ug/mL versus 8.38 ug/mL (p<0.001), and kynurenine levels were significantly higher, 0.453 ug/mL versus 0.36 2ug/mL (p<0.001) in WWH versus WWoH (**Extended Data Fig. 7 and Extended Data Tables 8 and 9**).

To further investigate the transfer of tryptophan or kynurenine to milk, we compared the quantitative levels in 92 women (21 WWoH and 71 WWH) who had both plasma and breast milk samples collected at the 4-month timepoint (**Extended Data Fig. 8**). Milk levels of tryptophan were 6.8% and 8.3% of the plasma levels for WWoH and WWH, respectively; this proportion did not differ significantly by maternal HIV infection (p=0.14, **Extended Data Fig. 8A and Extended Data Table 8**). A similar trend was observed for kynurenine with breast milk levels at 32.4% and 36.6% of the corresponding plasma levels for WWoH and WWH, respectively (p=0.42, **Extended Data Fig. 8B and Extended Data Table 8**). Altogether, these results suggest that the decreased levels of tryptophan and increased levels of kynurenine observed in the milk of WWH reflect plasma levels as opposed to selective transfer to the milk or altered local metabolism.

### Identification of ddhC, a free base of the naturally occurring antiviral ribonucleotide, ddhCTP

**Figure 4:**
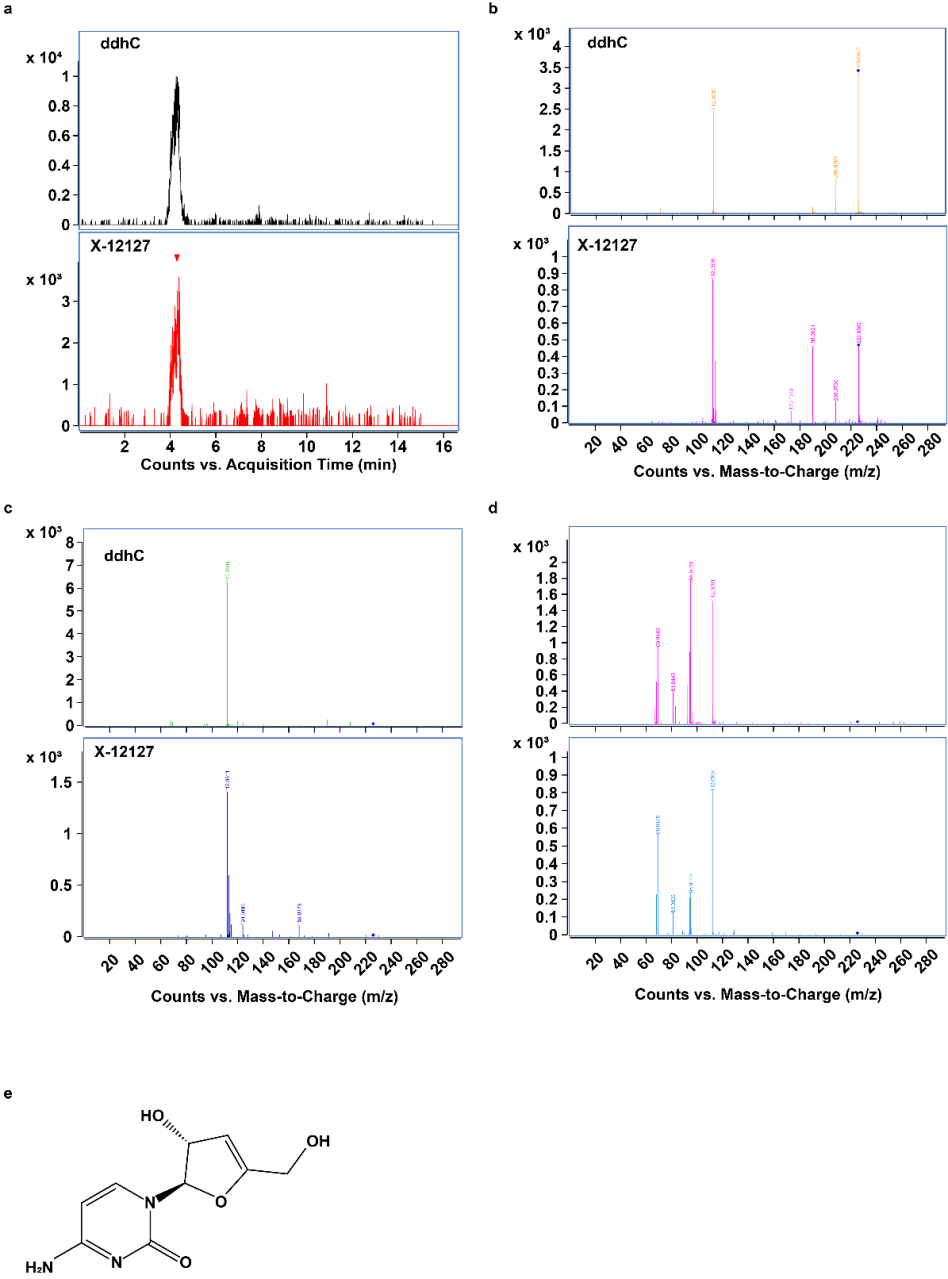
Identification of unknown compound X-12127 as 3’-deoxy-3’,4’-didehydro-cytidine (ddhC). a) Comparison of retention time (RT) for X-12127 and ddhC standard. b-d) MS/MS spectra of X-12127 and ddhC standard obtained on an Agilent 6546 LC/Q-TOF MS system in the positive targeted MS/MS scan mode by varying the collision energy (CE) at 5 ev (b), 20 ev (c), and 40 ev (d). e) Chemical structure of 3’-deoxy-3’,4’-didehydro-cytidine (ddhC).

Levels of an unknown compound, initially labeled X-12127, were significantly elevated in WWH compared to WWoH at all study visits except immediately postpartum (Figure 2B and **Extended Data Table 2**). Given the robustness of this marker, we sought to better characterize this compound by mass spectrometry. The compound, with an m/z value of 226.0824 in positive ionization mode, was analyzed using tandem MS/MS, revealing a cytosine fragment and two water loss events, suggesting the presence of two OH groups and a composition similar to 2’-deoxycytidine (dC) minus two hydrogen atoms (Figure 4). Structural similarity exploration and predicted fragmentation indicated 3’-deoxy-3’,4’-didehydro-cytidine (ddhC) as a likely candidate. This was confirmed through standard acquisition and extensive comparison of fragmentation patterns at different collision energies, leading to the definitive identification of the compound as ddhC (Figure 4E).

### Validation of milk metabolomic signature of HIV in a healthier cohort of WWH

To assess the generalizability of these findings, global metabolomics profiling was performed on the milk from a previously described cohort of 22 WWH and 25 WWoH from Haiti (Table 1)^43^. Notably, these women were on antiretroviral therapy with a mean CD4 count of 541 cells/mm^3^ and milk was sampled at a single visit during the first 6 months of lactation (median 45 days). Regression estimates of the associations between HIV status and the metabolites from the main and validation cohorts were strongly correlated (r=0.386, p<0.001 for all metabolites; r=0.828, p<0.001 when only the metabolites observed to be significantly associated with HIV status in the ZEBS cohort were included; **Extended Data Fig. 9**). Two metabolites were significantly increased in the milk of WWH, cytosine (p < 0.001) and a different unknown metabolite, X-12193 (p = 0.054) (**Extended Data Table 10**). Tryptophan trended lower and kynurenine trended higher in the milk of WWH in the Haiti cohort as observed in the main cohort, although neither reached statistical significance. Notably, the KT log-ratio was significantly higher in the milk of WWH (unadjusted p = 0.05).

## Discussion

Approximately 1.3 million children are born to WWH annually, and despite maternal antiretroviral therapy, these children continue to experience differences in immune function, growth and cognition. In this study, we find that the milk of WWH is characterized by decreased levels of tryptophan and perturbations of tryptophan catabolism via the kynurenine pathway. These interferon-inducible metabolic alterations may serve as a common denominator to explain some of the increased morbidity and mortality experienced by CHEU.

Normal immune function depends on the regulation of key components of innate and adaptive immunity in lymphoid tissues. Interplay between antigen presenting cells such as macrophages and dendritic cells are critical to host defense by maintaining mucosal barriers and attenuating inflammation. Immune dysregulation mediated by cellular immunity is associated with alterations in indoleamine 2,3-dioxygenase and tryptophan catabolism in individuals with HIV^29^. Chronic viral infections are also known to alter tryptophan catabolism^31^. Recent studies of post-acute sequelae of SARS-CoV-2 infection (PASC) demonstrate that individuals with long COVID have decreased circulating serotonin levels secondary to viral RNA-induced type I interferons reducing tryptophan uptake through the gut epithelium^42^. Additionally, a tryptophan deficient diet is sufficient to lead to circulating tryptophan and serotonin deficiency in a mouse model^42^. Tryptophan starvation appears to be part of the innate immune response to bacterial and parasitic infections^44^. Tryptophan catabolism, much of which is carried out by intestinal microbes, serves an essential role in physiological homeostasis and immune regulation and can rapidly be adapted in the stress response^32^. HIV infection may stimulate IDO-1 activity through virally associated increases in type I and II interferons and microbial products. Multiple tryptophan metabolites are endogenous ligands of the aryl hydrocarbon receptor (AHR), which serves as an environmental sensor that integrates immune responses^45^. Thus, deficiency or perturbation of tryptophan catabolites can lead to alterations of the gut microbiota and immunity by alterations in the IDO1-AHR axis^46^. Importantly, T helper cell subsets including T_H_17 and regulatory T cells express AHR, so alterations in these pathways may alter immune homeostasis and immune development in children of women with HIV^45^. In the context of growth, tryptophan serves as a limiting amino acid for protein metabolism. Additionally, alterations in tryptophan catabolism have been associated with stunting^38,39^. Cognitive effects of tryptophan depletion are thought to be due in part to decreased availability of serotonin as serotonin is critical for neuronal differentiation, migration, and synapse formation during embryologic development^46^. Altered tryptophan catabolism may also explain some of the neurocognitive deficits in children of WWH by leading to increases in neurotoxic metabolites, such as quinolinic acid, and decreases in neuroprotective metabolites, such as kynurenic acid^30^. Kynurenine and quinolinic acid are elevated in the cerebrospinal fluid of people with HIV and correlate with the severity of HIV-associated neurocognitive disorder^31,47^.

Type I interferons also lead to the production of 3’-deoxy-3’,4’-didehydro-cytidine triphosphate (ddhCTP), an innate antiviral compound produced by the interferon-inducible protein *viperin* (*v*irus *i*nhibitory *p*rotein, *e*ndoplasmic *r*eticulum-associated, *in*terferon-inducible)^40^. ddhC, which is highly elevated in the milk of WWH, is the free base of this naturally occurring antiviral ribonucleotide and is also being investigated as a biomarker for acute viral infection^48 49^. ddhCTP has direct antiviral properties and acts as a chain terminator for the RNA-dependent RNA polymerases from multiple members of the Flavivirus genus^40^, Zika Virus, and likely SARS-CoV-2^49–51^. Furthermore, HIV-1 infection of macrophages induces *viperin*-mediated inhibition of viral replication^52^. *Viperin* is a multifunctional protein and product of an ancient gene that is part of the radical *S-*adenosyl-L-methionine (SAM) superfamily of enzymes^51^. The metabolic pathways, products, and serum cytokine associations of *viperin* are being elucidated^49^. Viperin is co-transcribed with cytidylate monophosphate kinase 2 (*CMPK2*) during interferon stimulation. It has been proposed that CMPK2 primarily functions to produce enough substrate for the viperin-mediated production of ddhCTP which catalyzes the conversion of cytidine triphosphate to ddhCTP.

Cytosine was also persistently elevated in the milk of WWH. Viral infection has been associated with alterations in host cytosine metabolism^53^ and the production of ddhCTP^54^. While ddhCTP is a viral chain terminator, host RNA and DNA polymerases are not affected by ddhCTP^40^. We found cytosine elevations in both our primary cohort and in our validation cohort. We hypothesize that the elevated levels of cytosine in the milk of WWH reflects breakdown of ddhCTP. The elevation of dimethylarginine alone and with the N-acetylated amino acids (N-acetylalanine and N-acetylserine) in our cohort is consistent with oxidative stress and increased protein catabolism. Our study does not discriminate symmetric versus asymmetric dimethylarginine. There is emerging data on symmetric arginine methyltransferases potentially playing an important role in immune cell development and homeostasis^55^. Alterations in T cell development may underpin the immunologic abnormalities seen in children of WWH.

These findings raise the question of whether replacement of tryptophan in the diet of children of WWH will lead to resolution of the immune, cognitive and growth perturbations experienced by this population. Breastfeeding remains the recommended form of infant feeding because health benefits outweigh the risk of HIV transmission. With effective maternal antiretroviral therapy, HIV transmission risk has decreased to the point where shared decision making about breastfeeding entered US guidelines in 2023. The decreased levels of milk tryptophan in the setting of chronic HIV infection are likely due to downregulation of tryptophan transporters in the maternal gut resulting in lower tryptophan plasma levels^42^. Whether the pro-inflammatory milieu of milk similarly affects infant tryptophan absorption is unknown. Prudence is also demanded as inflammatory imprinting from the milk of WWH might drive tryptophan catabolism down the IDO pathway and lead to subsequent buildup of neurotoxic tryptophan metabolites in the developing infant. That CHEU infants display perturbed biomarkers of monocyte activation including elevations in interferon-inducible cytokines is supportive of this possibility^15–18^. Careful study of tryptophan replacement in the setting of chronic viral inflammation in animal models will be necessary to ensure that replacement will result in beneficial effects. If replacement alone is insufficient, then replacement with agents to modulate the kynurenine pathway such as IDO-inhibitors, which are in development, may be necessary^31^.

The primary strength of this investigation is the longitudinal profiling of almost 1600 milk samples from a deeply characterized cohort from a randomized clinical trial. The dense and consistent sampling over the first 18 months postpartum allows us to identify both temporally specific and persistent effects of maternal HIV infection. That this large study utilizes samples taken in the pre-antiretroviral therapy era is both a blessing and limitation. It is often difficult to deconvolute the profound metabolic signatures of different antiretroviral agents and HIV infection itself, which makes analysis of pure disease signatures more complex in the setting of antiretroviral therapy. However, the strength and robustness of the signatures for HIV infection observed in the present study are likely to some extent due to the severity of disease progression in the absence of effective antiretroviral therapy; maternal CD4 counts, viral loads, and tragically, mortality are all abundant evidence to this point. Growing evidence suggests that antiretroviral therapy reduces but does not fully ameliorate the chronic inflammation associated with HIV infection. Neither, unfortunately, does maternal antiretroviral therapy fully normalize the adverse health outcomes of CHEU. Consistent with these points, the metabolomic signature in the milk of WWH is still observed in the validation cohort of women on antiretroviral therapy with higher CD4 counts, suggesting that the underlying mechanisms likely remain relevant in modern settings with universal antiretroviral therapy.

### Conclusion

**Figure 5:**
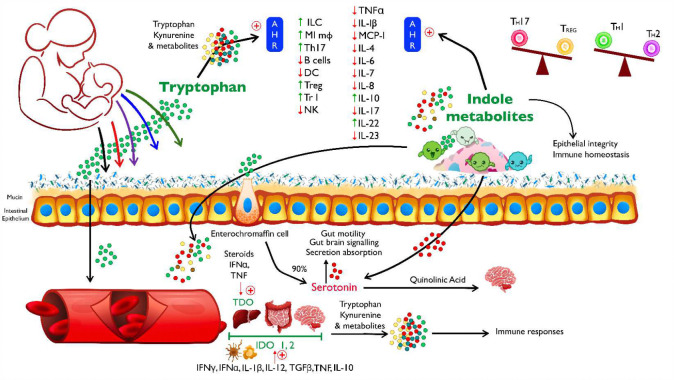
Proposed mechanism. Chronic viral inflammation from HIV infection leads to elevation of type I interferons, which in turn modulates intestinal absorption of tryptophan. Decreased tryptophan absorption leads to decreased circulating tryptophan and lower tryptophan levels in the milk of WWH in the setting of a proinflammatory milieu. Since milk is the source of tryptophan for the infants of WWH while they are exclusively breastfeeding, tryptophan levels and alterations in tryptophan catabolism may contribute to the immunologic, growth and cognitive differences in CHEU.

We identified a robust and persistent metabolomic signature in the milk of WWH comprising the kynurenine/tryptophan axis as well as dimethylarginine, cytosine, and ddhC, the free base of a recently discovered antiviral molecule, ddhCTP, that spans the first 18 months of lactation. This signature is consistent with a model (see Figure 5) in which chronic viral inflammation associated with maternal HIV infection leads to induction of the type I interferon response. Virally induced elevation of type I interferons decreases transport of tryptophan across the gastrointestinal wall, thereby leading to systemic depletion of tryptophan and subsequent reduction of tryptophan levels in the milk of WWH. Since human milk is the only source of nutrition for exclusively breastfed infants, maternal tryptophan deficiency and chronic immune activation during gestation and lactation may drive subsequent serotonin deficiency and altered tryptophan catabolism in children of women with HIV. Taken together, these findings provide a mechanism that potentially explains the widely reported immunologic, neurocognitive and growth abnormalities associated with CHEU infants and offer intriguing opportunities for selective interventions to ameliorate this substantial global burden.

## Methods

### Study Design

#### Clinical Trial Group and parent trial study intervention:

The Zambia Exclusive Breastfeeding Study (ZEBS) conducted in Lusaka, Zambia between 2001 and 2008 investigated a feeding strategy for WWH recommended by the World Health Organization at that time. The strategy, intended to decrease infant infection with HIV during an era prior to widespread availability of antiretroviral medications, was to rapidly wean infants from breastfeeding after 4 months of lactation. In ZEBS, WWH were randomized to either breast feed until usual breastfeeding cessation or to rapidly wean at 4 months. At the 4- and 4.5-month timepoints, only milk samples from women who reported they were actively still breastfeeding were included in the analysis. Almost all women received only single dose nevirapine for the prevention of vertical transmission as was standard of care at the time. Mother/infant pairs were followed for 24 months postpartum. The clinical characteristics of the women included in this sub study are given in Table 1.

#### Sample collection:

Whole breast milk and plasma samples were stored at −80C as previously published^56^.

#### Sample Selection and Comparison Groups:

##### Breast milk sample selection for the global metabolomics panel:

Longitudinal breast milk samples spanning the first 24 months of lactation from women living without (N=38) and with HIV (N=288) were selected. Samples were selected from all 38 women living without HIV (WWoH) included in the trial, 35 of their infants survived (279 samples) and 3 infants died (9 samples). A stratified sample of WWH were selected for inclusion by known infant outcomes for this metabolomics study. A random sample of 76 women with infants who did not experience HIV infection and who survived (N=76; 396 samples) or died (N=78; 193 samples), and women whose infants tested positive for HIV between 1 week and 1 month of life (N=53; 221 samples) or after 1 month of life (N=81; 401 samples).

##### Breast milk and plasma sample selection for the quantitative KT panel:

A random subset of 21 women without HIV and 80 women with HIV, 20 each from the groups above, with paired maternal plasma and milk samples at the 4-month timepoint were selected for the quantitative KT panel. The 4-month timepoint was selected because this was the first plasma collection during lactation.

##### Haiti validation cohort:

Milk samples from WWoH (n=25) and WWH (n=22) from a previously described cross-sectional study were used as an independent validation cohort^43^. In contrast to the main study cohort, these women were primarily on antiretroviral therapy and had relatively minor disease progression, most with CD4 counts above 350.

##### Metabolomics on the ZEBS Cohort:

Global metabolomics was performed on 1599 whole human milk samples collected longitudinally using ultra high-performance liquid chromatography/tandem mass spectrometry by Metabolon Inc. according to published methods^57–59^.

##### Sample Preparation:

Samples were prepared using the automated MicroLab STAR^®^ system from Hamilton Company. Several recovery standards were added prior to the first step in the extraction process for QC purposes. To remove protein, dissociate small molecules bound to protein or trapped in the precipitated protein matrix, and to recover chemically diverse metabolites, proteins were precipitated with methanol under vigorous shaking for 2 min (Glen Mills GenoGrinder 2000) followed by centrifugation. The resulting extract was divided into multiple fractions: two for analysis by two separate reverse phase (RP)/UPLC-MS/MS methods with positive ion mode electrospray ionization (ESI), one for analysis by RP/UPLC-MS/MS with negative ion mode ESI, one for analysis by HILIC/UPLC-MS/MS with negative ion mode ESI, while the remaining fractions were reserved for backup. Samples were placed briefly on a TurboVap^®^ (Zymark) to remove the organic solvent. The sample extracts were stored overnight under nitrogen before preparation for analysis.

##### QA/QC:

Several types of controls were analyzed in concert with the experimental samples: a pooled client matrix (CMTRX) sample generated by taking a small volume of each experimental sample served as a technical replicate throughout the data set; extracted water samples served as process blanks; and a cocktail of QC standards that were carefully chosen not to interfere with the measurement of endogenous compounds were spiked into every analyzed sample, allowed instrument performance monitoring and aided chromatographic alignment.

##### UltraHigh Performance Liquid Chromatography-Tandem Mass Spectroscopy (UPLC-MS/MS):

All methods utilized a Waters ACQUITY ultra-performance liquid chromatography (UPLC) and a Thermo Scientific Q-Exactive high resolution/accurate mass spectrometer interfaced with a heated electrospray ionization (HESI-II) source and Orbitrap mass analyzer operated at 35,000 mass resolution^58^. The dried sample extracts were then reconstituted in solvents compatible to each of the four methods. Each reconstitution solvent contained a series of standards at fixed concentrations to ensure injection and chromatographic consistency. One aliquot was analyzed using acidic positive ion conditions, chromatographically optimized for more hydrophilic compounds (PosEarly). In this method, the extract was gradient eluted from a C18 column (Waters UPLC BEH C18–2.1×100 mm, 1.7 μm) using water and methanol, containing 0.05% perfluoropentanoic acid (PFPA) and 0.1% formic acid (FA). Another aliquot was also analyzed using acidic positive ion conditions; however, it was chromatographically optimized for more hydrophobic compounds (PosLate). In this method, the extract was gradient eluted from the same aforementioned C18 column using methanol, acetonitrile, water, 0.05% PFPA and 0.01% FA and was operated at an overall higher organic content. Another aliquot was analyzed using basic negative ion optimized conditions using a separate dedicated C18 column (Neg). The basic extracts were gradient eluted from the column using methanol and water, however with 6.5mM Ammonium Bicarbonate at pH 8. The fourth aliquot was analyzed via negative ionization following elution from a HILIC column (Waters UPLC BEH Amide 2.1×150 mm, 1.7 μm) using a gradient consisting of water and acetonitrile with 10mM Ammonium Formate, pH 10.8 (HILIC). The MS analysis alternated between MS and data-dependent MS^n^ scans using dynamic exclusion. The scan range varied slightly between methods but covered 70–1000 m/z. Raw data were uploaded to a publicly available database, see data availability below.

##### Data Analysis:

Compounds were identified by comparison to library entries of purified standards or recurrent unknown entities based on authenticated standards that contains the retention time/index (RI), mass to charge ratio (*m/z)*, and fragmentation data. Biochemical identifications are based on three criteria: retention index within a narrow RI window of the proposed identification, accurate mass match to the library +/− 10 ppm, and the MS/MS forward and reverse scores between the experimental data and authentic standards. *Metabolite Quantification and Data Normalization:* Peaks were quantified using area-under-the-curve. A data normalization step was performed to correct variation resulting from instrument inter-day tuning differences. Essentially, each compound was corrected in run-day blocks by registering the medians to equal one (1.00) and normalizing each data point proportionately.

##### Metabolomics on the Haiti Cohort:

Frozen human milk samples from the Haiti Cohort were processed by Metabolon, Inc using the CMTRX made from the ZEBS Cohort in order for the data to be merged. Instrument variability was determined by calculating the median relative standard deviation (RSD) for the internal standards that were added to each sample prior to injection into the mass spectrometers. Overall process variability was determined by calculating the median RSD for all endogenous metabolites present in 100% of the Client Matrix samples, which are technical replicates of pooled client samples.

##### Identification of ddhC:

The independent identification of ddhC was conducted at the Northwest Metabolomics Research Center, University of Washington. Breast milk samples were processed using a protein precipitation method. Briefly, 250 μL methanol was added to 50 μL breast milk, vortexed, stored at −20 °C for 20 min, centrifuged at 18,000 × g for 15 min at 4 °C, and 150 μL of the supernatant was collected. Samples were dried in a Vacufuge at 30 °C and reconstituted in 500 μL of LC-matching solvent before LC-MS analysis.

MS analysis was performed using an Agilent 6546 LC/Q-TOF MS using HILIC chromatography. Accurate mass analysis identified the molecular formula of X-12127 as C9H11N3O4, based on the [M+H]+ ion. Tandem MS/MS with a collision energy ramp of 5–40 V revealed its fragmentation patterns. While no database matches were found, the presence of a cytosine fragment suggested the metabolite might be ddhC. This identification was confirmed by matching the experimental MS/MS spectrum and retention time of [M+H]+ with a ddhC standard, and the identification was finalized using a parallel analysis of a ddhC reference standard with the study samples.

##### Quantitative KT Panel:

Plasma and whole breast milk samples were analyzed for Tryptophan and Kynurenine by LC-MS/MS (Metabolon Method TAM220: “LC-MS/MS Method for The Determination of 15 Metabolites in Human Plasma (GFR-Panel)”. The method was adapted to analyze whole breast milk samples. Briefly, the plasma and whole milk samples were extracted with an organic solvent containing isotopically labeled internal standards (Tryptophan-d5, CIL Catalog# DLM-1092-0 and Kynurenine-d6, CIL Catalog# DLM-7842-0), injected on the Agilent 1290/SCIEX QTrap 5500 LC MS/MS system with a BEH Amide UHPLC column operating in positive mode using electrospray ionization, and the peak area of each analyte product ions measured against the peak area of the product ions of the corresponding internal standards. Quantitation was performed using a weighted linear least squares regression analysis generated from fortified calibration standards with ranges of 0.0250–10.0ug/mL for L-Kynurenine and 0.200–80.0ug/mL for L-Tryptophan that were generated immediately prior to the run (Tryptophan, Sigma Catalog# 93659 and Kynurenine, Sigma Catalog# 61250).

##### Analysis:

Principal coordinates analysis and permutational multivariate analysis of variance (PERMANOVA) were performed using Euclidean distances calculated on normalized metabolite abundances. Linear mixed effects models of the form metabolite ~ HIVExposure*Visit + (1 | PID) where *HIVExposure* is an indicator variable for WWH versus WWoH, *Visit* is a categorical variable for the study visit, and *PID* is the unique participant identifier were used to identify differentially abundant metabolites as well as differences in KT log-ratio. Estimated marginal means were used to conduct the post hoc comparisons either averaged or stratified by study visit. Results are reported as t-ratios, which are the estimates of the regression coefficients divided by their standard error. For the quantitative KT panel data, comparisons were performed using a Kruskal-Wallis (for more than two categories) or Welch’s t (for two categories) test. Spearman rank correlation was used to compare global and quantitative KT data, and to compare metabolite abundances with maternal CD4 counts and viral loads.

Random forests classification models were constructed separately for each study visit with *HIVExposure* as the outcome. A two-step approach was utilized to select both the optimal number of features as well as the specific features used for each model. In the first step, one hundred forests each comprising 10,000 trees were built to obtain feature importance values calculated as mean decrease in accuracy. In the second step, tenfold cross-validation with a sequentially reduced number of features was then used to identify the optimal number of features to be used. Finally, a sparse model was constructed for each study visit containing the optimal number of metabolites calculated in step 2 (up to a maximum of twenty features to aid interpretability), with features selected by the highest importance as calculated in step 1. Model accuracy, sensitivity, specificity, and Matthew’s correlation coefficient were calculated from the out-of-bag error estimate for the final sparse model.

All statistical analyses were conducted in the R statistical environment (version 4.1.3). A full list of all R packages used in the analyses is given in Appendix A. All p-values were adjusted for multiple comparisons using the Benjamini-Hochberg false discovery rate method, and a cutoff of p<0.05 was accepted as significant.

## Figures and Tables

**Table 1:** Baseline Characteristics of Study Cohorts

Baseline Characteristics of Zambia Exclusive Breastfeeding Study Cohort			
Value (median (Standard Deviation))	WWoH	WWH	p-value
N	38	288	
Maternal Age (years), median (SD)	25 (7)	26 (5)	0.291
Parity	2.3 (1.9)	2.5 (1.7)	0.510
Log10 HIV RNA, median (SD)	NA	4.81 (0.76)	
Maternal CD4 Count (cells/mm^3^)			
Median (SD)	819 (246)	276 (185)	<0.001
<200 cells/mm^3^ (n (%))	0 (0.0)	94 (32.6)	
200–349 cells/mm^3^ (n (%))	2 (5.3)	98 (34.0)	
>350 cells/mm^3^ (n (%))	36 (94.7)	96 (33.3)	
Maternal CD8 Count (cells/mm^3^), median (SD)	503 (233)	710 (345)	0.001
Maternal CD3 Count (cells/mm^3^), median (SD)	1392 (434)	1082 (450)	<0.001
Caesarean Section (%)	0 (0.0)	5 (1.7)	NA
Gestational Age at Delivery (weeks), median (SD)	38.1 (5.4)	38.3 (4.1)	
Infant Birth Weight (kg), median (SD)	3.20 (1.24)	3.00 (0.49)	0.005
Infant Male Sex = n (%)	13 (34.2)	143 (49.7)	0.106
12-month CD4 Count (cells/mm^3^), median (SD)	868 (349)	302 (298)	<0.001
Child death (%)	3 (7.9)	145 (50.3)*	NA
Maternal death (%)	0 (0.0)	36 (12.5)	0.042
Baseline Characteristics of Haiti Cohort			
Value (median (Standard Deviation))	WWoH	WWH	p-value
N	25	22	
Maternal Age (years), median (SD)	27 (7)	31 (6)	0.119
Maternal BMI, median (SD)	21.4 (2.8)	25.0 (3.8)	0.028
Parity	3.0 (1.9)	3.3 (1.8)	0.565
Log10 HIV RNA, median (SD)		1.59 (1.45)	NA
Plasma HIV RNA (copies/mL)			NA
<1000 (n (%))		15 (68.2)	
>1000 (n (%))		7 (31.8)	
Maternal CD4 Count (cells/mm^3^)			NA
Median (SD)	NA	541 (271)	
<200 cells/mm^3^ (n (%))		1 (4.5)	
200–349 cells/mm^3^ (n (%))		3 (13.6)	
>350 cells/mm^3^ (n (%))		18 (81.8)	
Maternal CD4 Count (cells/mm^3^)			NA
<350 cells/mm^3^ (n (%))		4 (18.2)	
>350 cells/mm^3^ (n (%))		18 (81.8)	
Caesarean Section (%)	1 (4.0)	5 (22.7)	0.138
Infant Age in Days, median (SD)	72 (57)	45 (46)	0.725

## Data Availability

All MS data is available at MetaboLights^60^: https://www.ebi.ac.uk/metabolights/index **MTBLS2307 UCLA-03-22PHML** (1599 breast milk samples) https://www.ebi.ac.uk/metabolights/MTBLS2307 **MTBLS2573 UCLA-06-23MD+** (50 breast milk samples) Metabolon https://www.ebi.ac.uk/metabolights/MTBLS2573 **Analysis code availability:** The analysis code is available at GitHub: https://github.com/fanli-gcb/BMM

## Appendix A (R packages used):

**Table:**

Package	Version	Use
vegan	2.6-2	PERMANOVA
lmerTest	3.1–3	Linear mixed effects models
lme4	1.1–30	Linear mixed effects models
emmeans	1.8.1–1	Estimated marginal means of linear mixed effects models
ranger	0.13.1	Random forests analyses

## List of Abbreviations

CHEU: Children whose mothers had HIV infection (“exposed”) but not infected with HIV
HEU: HIV-exposed uninfected
HIV: Human Immunodeficiency Virus
SD: Standard Deviation
WWH: Women with HIV
WWoH: Women without HIV
ZEBS: Zambia Exclusive Breastfeeding Study
WWoH: Women without HIV
WWH: Women with HIV
SD: Standard deviation
BMI: Body Mass Index
